# E-cadherin expression phenotypes associated with molecular subtypes in invasive non-lobular breast cancer: evidence from a retrospective study and meta-analysis

**DOI:** 10.1186/s12957-017-1210-8

**Published:** 2017-08-01

**Authors:** Jiang-Bo Liu, Chen-Yi Feng, Miao Deng, Dong-Feng Ge, De-Chun Liu, Jian-Qiang Mi, Xiao-Shan Feng

**Affiliations:** 1 Department of General Surgery, First Affiliated Hospital, College of Clinical Medicine, Henan University of Science and Technology, Luoyang, 471003 China; 2 Henan Key Laboratory of Cancer Epigenetics, Cancer Institute, First Affiliated Hospital, College of Clinical Medicine, Henan University of Science and Technology, Luoyang, 471003 China; 3 Department of Pathology, First Affiliated Hospital, College of Clinical Medicine, , Henan University of Science and Technology, Luoyang, 471003 China

**Keywords:** E-cadherin, Breast cancer, Immunohistochemistry, Molecular subtypes, Meta-analysis

## Abstract

**Background:**

This retrospective study and meta-analysis was designed to explore the relationship between E-cadherin (E-cad) expression and the molecular subtypes of invasive non-lobular breast cancer, especially in early-stage invasive ductal carcinoma (IDC).

**Methods:**

A total of 156 post-operative cases of early-stage IDCs were retrospectively collected for the immunohistochemistry (IHC) detection of E-cad expression. The association of E-cad expression with molecular subtypes of early-stage IDCs was analyzed. A literature search was conducted in March 2016 to retrieve publications on E-cad expression in association with molecular subtypes of invasive non-lobular breast cancer, and a meta-analysis was performed to estimate the relational statistics.

**Results:**

E-cad was expressed in 82.7% (129/156) of early-stage IDCs. E-cad expression was closely associated with the molecular types of early-stage IDCs (*P* < 0.050); moreover, the molecular subtypes were an independent factor influencing E-cad expression in early-stage IDCs. A total of 12 observational studies (including our study) were included in the meta-analysis. The meta-analytical results show a significantly greater risk of E-cad expression loss in triple-negative breast cancer (TNBC) than in other molecular subtypes (TNBC vs. luminal A: RR = 3.45, 95% CI = 2.79–4.26; TNBC vs. luminal B: RR = 2.41, 95% CI = 1.49–3.90; TNBC vs. HER2-enriched: RR = 1.95, 95% CI = 1.24–3.07).

**Conclusions:**

Early-stage IDCs or invasive non-lobular breast cancers with the TNBC molecular phenotype have a higher risk for the loss of E-cad expression than do tumors with non-TNBC molecular phenotypes, suggesting that E-cad expression phenotypes were closely related to molecular subtypes and further studies are needed to clarify the underlying mechanism.

**Electronic supplementary material:**

The online version of this article (doi:10.1186/s12957-017-1210-8) contains supplementary material, which is available to authorized users.

## Background

Breast cancer is the most common malignancy in women worldwide, with approximately 246,660 new cases occurring among women in the USA in 2016 [1]. The survival of breast cancer patients has been significantly improved in the past decades; however, invasion and metastasis still result in many deaths in patients with advanced breast cancer [1, 2]. Conventional prognostic factors, such as tumor staging and grading, do not always efficiently estimate clinical outcomes in individual breast cancer patients because of the complex characteristics of the disease [3, 4]. Therefore, the discovery of molecular markers to aid in tumor-type stratification and breast cancer surveillance is critical [5, 6].

Molecular subtypes are closely related to the patterns of metastasis and natural courses of breast cancer, and specific treatment models for different molecular subtypes of breast cancer can improve the prognosis of patients with the disease [7, 8]. E-cadherin (E-cad) is a calcium-dependent epithelial transmembrane glycoprotein that mediates cell-to-cell adhesion and helps maintain the morphological integrity of epithelial cells [9]. Typically, a loss of E-cad expression occurs when cancer cells undergo an epithelial-mesenchymal transition (EMT) [10, 11]. The loss of E-cad expression has been found to be significantly associated with a lack of estrogen receptor (ER) expression, the expression of cytokeratins 5/6 and/or epidermal growth factor receptor (EGFR), and a basal-like phenotype (or triple-negative breast cancer, TNBC) in breast cancer [12, 13]. Studies have also confirmed that many breast cancers in which E-cad expression is lost have a lobular morphology and show aggressive invasion and metastasis [14, 15]. Invasive ductal carcinomas (IDCs) encountered in clinical practice usually have typical molecular subtypes and show a low frequency of E-cad expression loss [12, 16, 17]. Moreover, molecular subtypes are critical for determining the direction of adjuvant systemic therapies for treating early-stage breast cancer and have important implications for patient care [8, 18]. However, there is little information clarifying the association between E-cad expression and the molecular subtypes of early-stage IDC.

In this study, we evaluated the expression of E-cad in a panel of early-stage (stage I and II) IDCs to assess the association of E-cad expression with the molecular subtypes and the clinical and molecular pathological characteristics of the disease to provide further evidence for use in evaluating the risk of recurrence and metastasis in patients. Furthermore, our study also investigated the association of E-cad expression and the molecular subtypes of invasive non-lobular breast cancer by performing a meta-analysis of published studies.

## Methods

### Retrospective study

#### Patient selection

This study was approved by the Medical Ethics Committee of the First Affiliated Hospital of Henan University of Science and Technology, and informed consent was obtained from all the patients involved with the collection of tissue samples. The inclusion criteria for the retrospective study were early-stage IDC, including stage I and II diseases, proved by pathology, and treated with radical surgical operation followed by endocrine therapy, chemotherapy, targeted therapy, and/or radiotherapy. Paraffin-embedded specimens were collected continuously from all patients with early-stage IDC (*n* = 156) who underwent surgical intervention and pathological examination from September 2011 to October 2014 in the First Affiliated Hospital of Henan University of Science and Technology. The clinical and pathological data of these 156 patients were obtained from the patients’ records retrospectively.

#### Immunohistochemical evaluation and definition of molecular subtypes

Immunohistochemistry (IHC) methods were performed using an ultrasensitive SP-IHC kit according to the manufacturer’s protocol (Maxim Biotech, Inc., Fuzhou, China). Anti-E-cad (clone 4A2C7), anti-ER (clone SP1), anti-progesterone receptor (PR) (clone SP2), anti-human epidermal growth factor receptor 2 (HER2) (clone EP3), and anti-Ki67 (clone MIB-1) mouse monoclonal antibodies (Maxim Biotech, Inc., Fuzhou, China) were used for the IHC detection of E-cad, ER, PR, HER2, and Ki67 expression. A 3,3-diaminobenzidine tetrahydrochloride (DAB) kit (Maxim Biotech, Inc., Fuzhou, China) was used to visualize the detected markers.

**Fig. 1:**
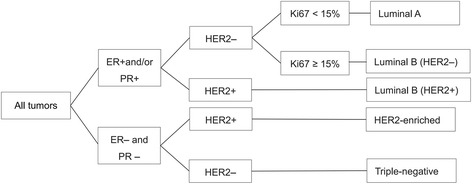
Classification algorithm for molecular subtyping [8, 19]

All IHC evaluations were performed independently by two pathologists. Samples were considered positive for ER and PR expression if ≥10% of the tumor cells showed positive nuclear staining. For Ki67, if ≥15% of the nuclei were stained, samples were classified as showing positive (high) expression. HER2 expression is located in breast cancer cell membranes, and according to the scoring system (0, 1+, 2+, and 3+) of the American Society of Clinical Oncology/College of American Pathologists clinical practice guidelines, a score of 3+ was considered to indicate HER2-positive samples. E-cad expression was considered positive if greater than or equal to 50% continuous membrane staining was present in the breast cancer cells, and negative or low expression if less than 50%, which was the median percentage observed in the included subjects. In this study, based on the St Gallen International Expert Consensus on primary therapy for early-stage breast cancer from 2013, all tumors were phenotyped into IHC molecular subtypes based on a surrogate immunopanel for ER, PR, HER2, and Ki-67 (Fig. 1) [8, 19].

#### Statistical analyses

The data were analyzed by using SPSS Statistics version 19.0 (SPSS Inc). Chi-square and Spearman rank correlation tests were performed to assess the differences and the associations, respectively, between the characteristics of different early-stage IDCs with E-cad expression phenotypes. A logistic regression analysis was performed to explore the independent and interactive relationships between E-cad expression and molecular pathological factors. Risk ratios (RRs) and 95% confidence intervals (CIs) were calculated for the explanatory factors and were adjusted for confounding factors, including ER, PR, HER2, and Ki67 expression, as well as molecular subtypes, histologic grade, tumor stage, nodal stage, and TNM stage. A *P* < 0.050 was statistically significant.

### Meta-analysis

#### Literature search and inclusion criteria

PubMed and the ISI Web of Knowledge database were searched in March 2016 to identify primary research publications reporting associations between E-cad expression and breast cancer molecular subtypes. The following search terms, including MeSH Terms, Title/Abstract keywords, or Text Word, were used for a comprehensive literature search: “breast neoplasm, breast cancer, or mammary cancer”; “E-cadherin, or E-cad”; and “molecular subtypes, basal-like, HER2-positive, HER2-enriched, triple negative, luminal, or TNBC”. The literature was restricted to peer-reviewed, full-text publications written in English and Chinese. Additionally, the reference lists of relevant studies were checked for possible additional publications missed in the search.

Inclusion criteria for this meta-analysis were as follows: (1) pathologically proven invasive non-lobular cases of breast cancer were studied; (1.1) the sample size included more than 50 IDC cases; (1.2) the percentage of IDC cases was more than 50%; (2) the molecular subtypes of breast cancer reported were based on an IHC expression analysis of surrogate markers; (2.1) TNBC and non-TNBC types were reported; (2.2) TNBC, luminal type, and HER2-enriched types were reported; or (2.3) TNBC, luminal A, luminal B, and HER2-enriched types were reported; (3) an explicit description of the IHC methodology and an evaluation of E-cad expression were presented; and (4) a description of the association between E-cad expression and the molecular subtypes of breast cancers was provided. Two researchers (JBL and CYF) independently read the titles and abstracts of the identified studies. If appropriate, the full text of the studies was then scrutinized to determine whether they met the selection criteria. For studies with overlapping populations, the most informative study was included.

#### Data extraction and methodological assessment

Two investigators (JBL and CYF) independently extracted the following data from the eligible articles: first author, year of publication, study location, recruitment period, sample size, histologic type, percentage of invasive lobular carcinoma (ILC) and IDC, stage of the disease, tissue processing protocol, antibodies and cutoff value used for E-cad expression, and molecular subtypes identified. Then, these two researchers independently evaluated the quality of each study per the scoring system (range 0 to 9) of the Newcastle-Ottawa Quality Assessment Scale (NOS) [20]. In this meta-analysis, a high-quality study was considered as one having a score of 6 or greater, while a low-quality study was regarded as one having a score of less than 6.

#### Statistical analyses

Review Manager (RevMan) 5.3 (Copenhagen: The Nordic Cochrane Centre, The Cochrane Collaboration, 2014) were used for the meta-analysis. We estimated the risk ratios (RRs) and 95% confidence intervals (CIs) for E-cad expression between different molecular subtypes of breast cancer. Between-studies heterogeneity was evaluated using the *I* ^2^ statistic (ranges 0 to 100%), with an *I* ^2^ statistic value greater than 50% indicating the presence of substantial heterogeneity. When *I* ^2^ was less than 50%, pooled RRs and 95% CIs were calculated using the Mantel-Haenszel method with fixed-effect models; otherwise, a random-effect model was adopted. Moreover, if significant heterogeneity existed, we took subgroup analysis to investigate potential sources of heterogeneity. A sensitivity analysis was also performed to evaluate the influence of individual studies on the outcome of the analysis. The publication bias of eligible studies was estimated using funnel plots: if an asymmetrical funnel was observed, a publication bias was considered to be present [21]. All statistical tests were 2-sided, and a *P* value <0.050 was considered significant.

## Results

### Retrospective study

#### E-cad expression and molecular subtypes of early-stage IDCs

**Fig. 2:**
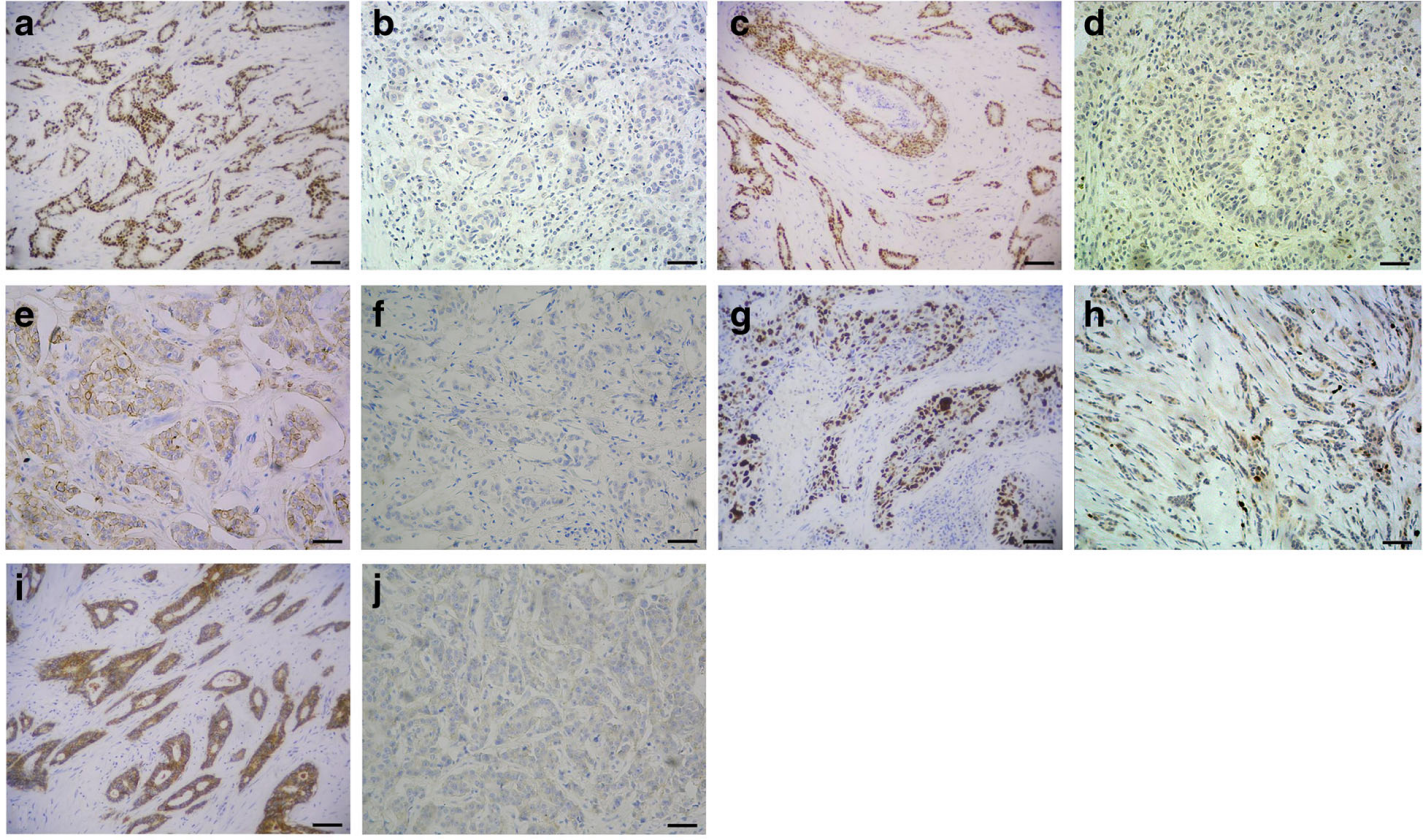
Expression of E-cad, ER, PR, HER2, and Ki67 in early stage IDC. **a** ER positive expression in nucleus of IDC. **b** ER negative expression in nucleus of IDC. **c** PR positive expression in nucleus of IDC. **d** PR negative expression in nucleus of IDC. **e** HER2 positive expression in membrane of IDC. **f** HER2 negative expression in membrane of IDC. **g** Ki67 positive expression in nucleus of IDC. **h** Ki67 negative expression in nucleus of IDC. **i** E-cad positive expression in membrane of IDC. **j** E-cad negative/low expression in membrane of IDC. Bar = 100 μm

The clinical and pathological data (histologic grade, tumor stage, nodal stage, and TNM stage) and IHC results for the 156 patients are summarized in Table 1. The positive expression rates for E-cad, ER, PR, HER2, and Ki67 expression were 82.7% (129/156), 66.7% (104/156), 47.4% (74/156), 17.3% (27/156), and 74.4% (116/156), respectively (Fig. 2). Based on the IHC results, the 156 cases of early-stage IDCs were classified into the following different molecular subtypes: 30 (19.2%) luminal A, 74 (47.4%) luminal B, 14 (9.0%) HER2-enriched, and 38 (24.4%) TNBC tumors.

**Table 1:** Clinic and pathological characteristics and IHC results of 156 early stage IDCs

Characteristics	No. of patients (*N* = 156) (%)
Age (years, mean ± SD)	52.8 ± 12.0
Histologic grade	
I	13 (8.3)
II	107 (68.6)
III	36 (23.1)
Tumor stage	
T1	77 (49.4)
T2	79 (50.6)
Nodal stage	
Negative	96 (61.5)
Positive	60 (38.5)
TNM stage	
I	50 (32.1)
II	106 (67.9)
E-cad	
Negative/low	27 (17.3)
Positive	129 (82.7)
ER	
Negative	52 (33.3)
Positive	104 (66.7)
PR	
Negative	82 (52.6)
Positive	74 (47.4)
HER2	
Negative	129 (82.7)
Positive	27 (17.3)
Ki67	
Negative	40 (25.6)
Positive	116 (74.4)
Molecular subtypes	
Luminal A	30 (19.2)
Luminal B	74 (47.4)
HER2-enriched	14 (9.0)
TNBC	38 (24.4)

*IHC* immunohistochemistry, *IDC* invasive ductal carcinoma of the breast, *TNBC* triple-negative breast cancer

#### Relationship between E-cad expression and early-stage IDC molecular subtypes

To understand the clinical importance of E-cad expression in early-stage IDCs, we compared the association of E-cad expression and the clinical and pathological features of the patients with the disease. A Spearman correlation analysis showed no significant association between E-cad expression and the clinical and pathological features of patients with early-stage IDCs. However, there was a statistically significant difference in the loss of E-cad expression among the different molecular subtypes of the disease (*P* = 0.049) (Table 2). We then separately compared E-cad expression in TNBC with that in the non-TNBC subtypes of early-stage IDCs, and the results show that TNBC tumors (31.6%) had a significantly higher rate of E-cad expression loss than what was observed in luminal A (10%, *P* = 0.033), luminal B (14.9%, *P* = 0.038), HER2-enriched (7.1%, *P* = 0.068), or non-TNBC (11.7%, *P* = 0.008) tumors.

**Table 2:** E-cadherin expression in early stage IDCs

Characteristics	No. of patients	E-cadherin		*χ* ^2^ (*P* value)
		Negative/low	Positive	
Histologic grade				
I	13	2	11	0.79 (0.673)
II	107	17	90	
III	36	8	28	
Tumor stage				
T1	77	15	62	0.50 (0.479)
T2	79	12	67	
Nodal stage				
Negative	96	20	76	2.17 (0.141)
Positive	60	7	53	
TNM stage				
I	50	11	39	1.13 (0.287)
II	106	16	90	
ER				
Negative	52	13	39	3.23 (0.073)
Positive	104	14	90	
PR				
Negative	82	16	66	0.59 (0.444)
Positive	74	11	63	
HER2				
Negative	129	24	105	0.43 (0.512)
Positive	27	3	24	
Ki67				
Negative	40	6	34	0.20 (0.655)
Positive	116	21	95	
Molecular subtypes				
Luminal A	30	3	27	7.85 (0.049)
Luminal B	74	11	63	
HER2-enriched	14	1	13	
TNBC	38	12	26	

*IDC* invasive ductal carcinoma of the breast, *TNBC* triple-negative breast cancer

#### Logistic regression analysis of clinical and pathological factors associated with E-cad expression loss in early-stage IDCs

To further explore the possible factors associated with E-cad expression loss (negative/low expression), we performed logistic regression analyses of the clinical and pathological factors, including ER, PR, HER2, and Ki-67 expression, as well as the molecular subtypes, histological grade, nodal stage, tumor stage, and TNM stage. The results show that molecular subtype was the only factor influencing E-cad expression loss in early-stage IDCs (RR = 1.779, 95% CI = 1.151–2.755, *P* = 0.010), suggesting that early-stage TNBC-IDC has an approximately twofold greater tendency to develop the loss of E-cad expression compared with expression loss in non-TNBC tumors (Table 3). We further analyzed the risk to develop E-cad expression loss between molecular subtypes. Similarly, the results show that TNBC tumors have a higher rate of E-cad expression loss compared with that of luminal subtypes (RR = 2.35, 95% CI = 1.19–4.61, *P* = 0.010), luminal B (RR = 2.12, 95% CI = 1.04–4.36, *P* = 0.040), and non-TNBC (RR = 2.48, 95% CI = 1.28–4.83, *P* = 0.007).

**Table 3:** Logistic regression analysis of clinic and pathological factors associated with E-cad expression loss in early stage IDCs

Variables	Estimate, B	Standard error	*Wald statistic*	*P* value^a^	*Risk ratio*	95% Confidence interval
Molecular subtypes	−0.577	0.223	6.714	0.010	1.779	1.151–2.755
Constant	3.080	0.654	22.203	0.000	0.046	

*IDC* invasive ductal carcinoma of the breast, *TNBC* triple-negative breast cancer

^a^Logistic regression analysis including ER, PR, HER2, Ki-67, molecular subtypes (TNBC vs. non-TNBC), histologic grade, nodal stage, tumor stage, and TNM stage

### Meta-analysis

#### Description of studies

**Fig. 3:**
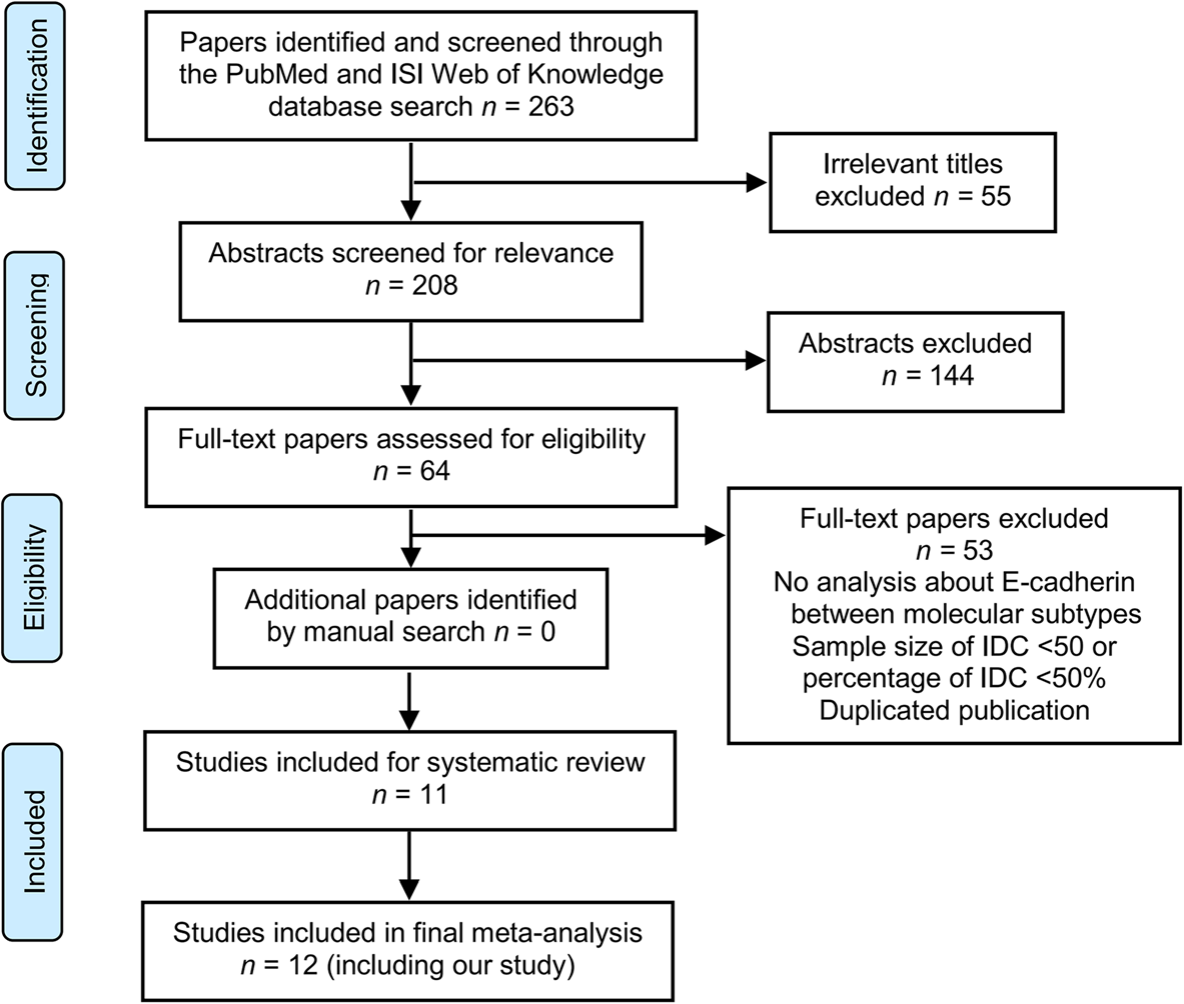
The PRISMA literature search flow chart

A total of 12 observational studies (including our retrospective study) from 10 medical centers were included in the meta-analysis [11, 12, 16, 17, 22–28]. Figure 3 illustrates the Preferred Reporting Items for Systematic Reviews and Meta-analyses (PRISMA) literature search flow chart [29]. The detailed characteristics of the 12 eligible studies are listed in Table 4. The median NOS quality score was 6 (range 5–7). Additional file 1: Table S1 provides detailed information about the quality assessment.

**Table 4:** Characteristics of the included studies in the meta-analysis

Studies	Country	Recruited period	No. of patients	% of ILC/IDC	Stage of disease	Tissue processing	Antibody/Cutoff	% of E-cad expression	Molecular subtypes	
									Non-TNBC	TNBC
Aleskandarany et al. [27]	UK	1990–1998	1035	0/70%	NR	TMA	C3865/>100 H-score^a^	50% (519/1035)	808 (luminal 652, HER2-enriched 156)	197
Choi et al. [11]	Korea	2009–2011	382 of 438	0/91% (397/438)	I–IV	TMA	NCH-38/10%	65% (247/382)	313 (luminal A 177, luminal B 96, HER2-enriched 40)	69
Jeong et al. [17]	Korea	1992–2006	492	0.6/97%	I–IV	TMA	SPM471/NR	85% (418/492)	363 (luminal 221, HER2-enriched 142)	102
Kashiwagi et al. [16]	Japan	2000–2006	574	0/83%	I–III	Whole slide section	NCH-38/≥30%	59% (336/574)	451	123
Liu et al. [25]	China	2000–2004	441	NR	I–IV	Whole slide section	Abcam/>10%	63% (276/441)	264 (luminal A 84, luminal B 90, HER2-enriched 90)	177
Mahler-Araujo et al. [12]	UK	NR	180 of 245	0/87% (156/180)	NR	TMA	HECD-1/≥50%	65% (142/217)	154	26
Pang et al. [26]	China	2001–2002	170	9%/78%	I–III	Whole slide section	Santa/>10%	36% (61/170)	129	41
Pomp et al. [28]	Switzerland	1991–2011	617	15%(87/565)/80% (454/565)	I–IV	TMA	EP700Y/>50%	88% (527/598)	478 (luminal 439, HER2-enriched 39)	120
Rakha et al. [22]	UK	1986–1998	1711 of 1722	NR/80% (1378/1722)	I–IV	TMA	HECD-1/>100 H-score	46% (795/1711)	1436	275
Sarrio et al. [23]	Spain	1993–2001	491	5/93%	NR	TMA	4A2C7/≥50%	45% (221/491)	399	64
Wu et al. [24]	China	2000–2006	382	2%/93%	I–IV	Whole slide section	4A2C7/≥50%	79% (301/382)	341 (luminal A 232, luminal B 45, HER2-enriched 64)	41
This study	China	2011–2014	156	0/100%	I–II	Whole slide section	4A2C7/≥50%	83% (129/156)	118 (luminal A 30, luminal B 74, HER2-enriched 14)	38

*ILC* invasive lobular carcinoma, *IDC* invasive ductal carcinoma of the breast, *TNBC* triple-negative breast cancer, *NR* not reported, *TMA* tissue microarray

^a^A semi-quantitative histochemical score method taking into account the intensity of staining and percentage of stained cells and giving a continuous measure (0–300) of marker expression [27]

For the included studies, which involved a total of 6631 patients (range 156–1711), the recruitment period for all patients was from 1986 to 2014. Ten of the 12 studies reported 70 to 100% of patients with IDC. All studies carried out IHC analyses for E-cad expression using formalin-fixed, paraffin-embedded tissue slides, and seven of these studies constructed tissue microarrays (TMAs). Of the included studies, the most frequently used antibodies against E-cad were monoclonal antibody 4A2C7 (*n* = 3), HECD-1 (*n* = 2), and NCH-38 (*n* = 2). The overall rate of E-cad expression loss in these studies was 40.1%, and the rates of E-cad expression loss for specific subtypes were 53.1% in TNBC (12 studies), 18.5% in luminal A (4 studies), 24.3% in luminal B (4 studies), and 26.6% in HER2-enriched (7 studies). A loss rate of 36.3% was reported in non-TNBC (12 studies).

#### Meta-analysis of the association of E-cad expression with molecular subtypes of invasive non-lobular breast cancer

**Fig. 4:**
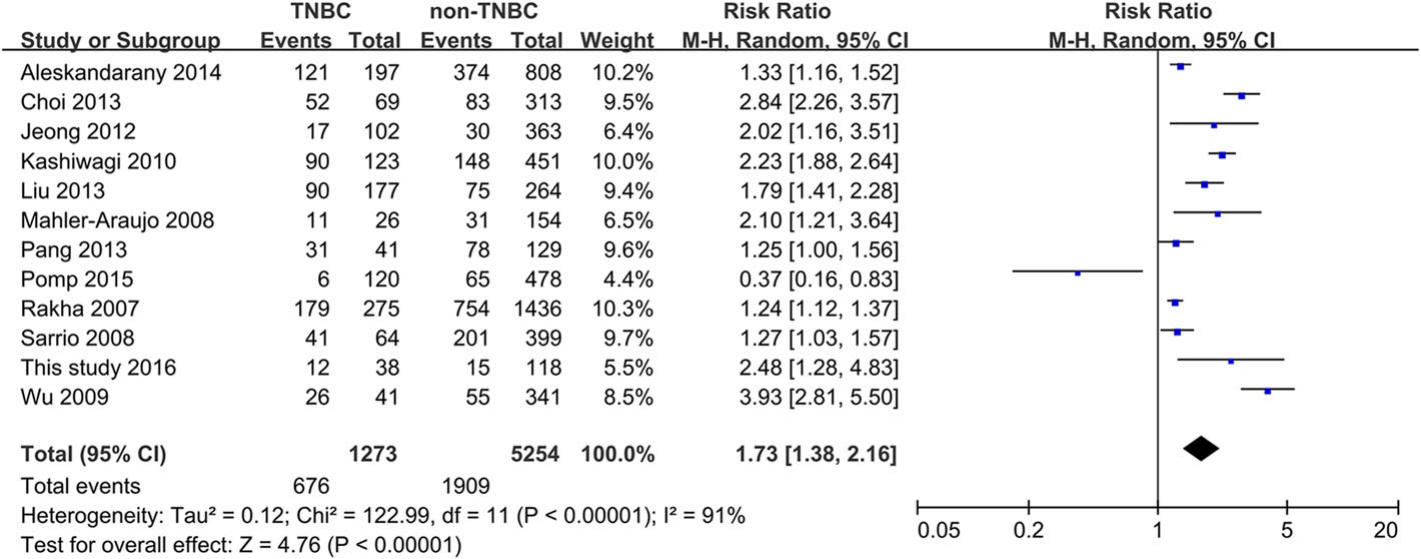
Comparison of E-cad expression loss between TNBC and non-TNBC tumors. *TNBC* triple-negative breast cancer

**Fig. 5:**
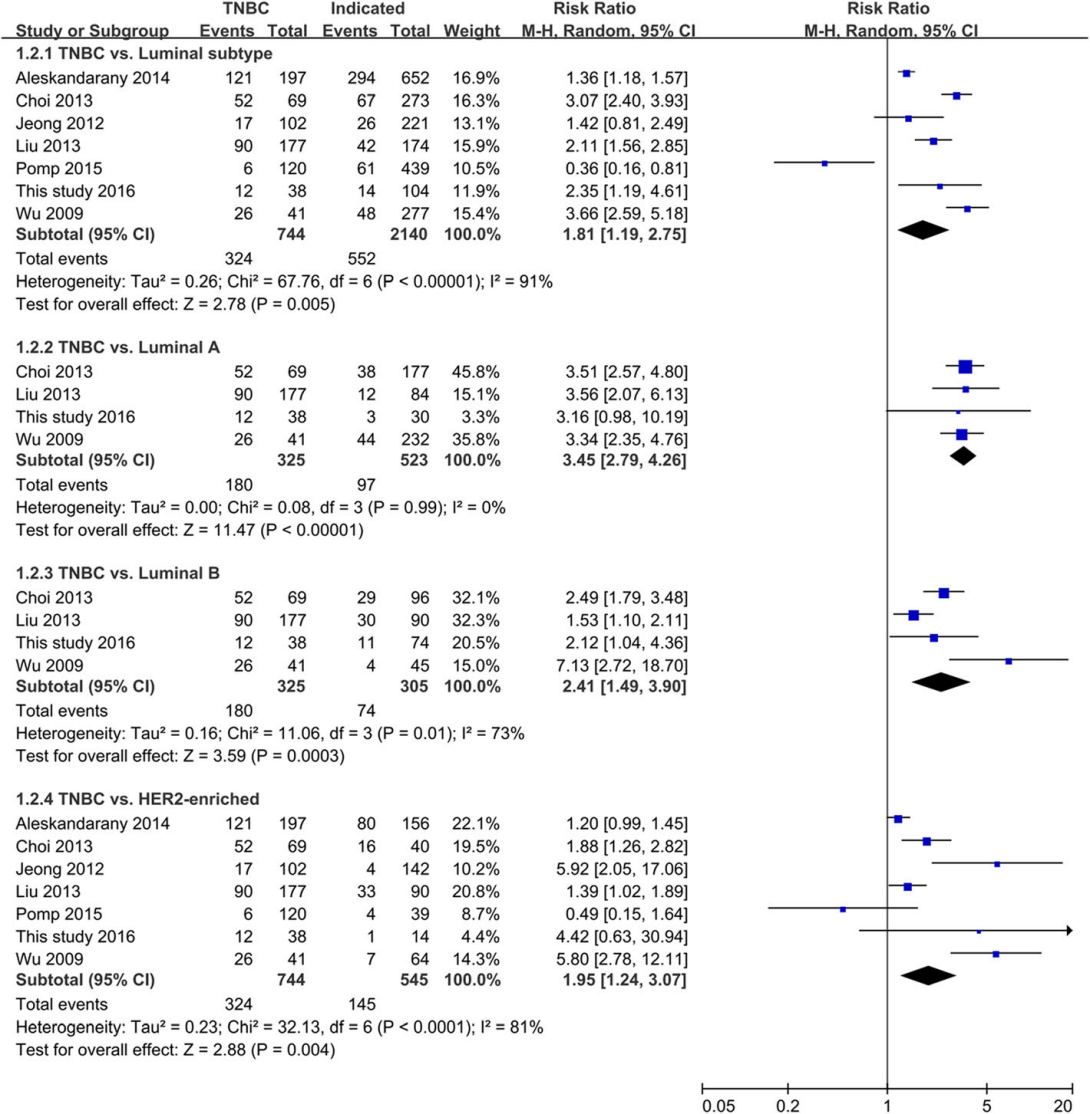
Comparison of E-cad expression loss between TNBC and luminal subtype and HER2-enriched tumors. *TNBC* triple-negative breast cancer

We performed pooled analyses with the available data on the pattern of E-cad expression in different molecular subtypes of invasive non-lobular breast cancers, and the results show significant difference in E-cad expression between the molecular subtypes of this disease (*P* < 0.010 in all cases between four molecular subtypes (4 studies), three molecular subtypes (7 studies), or two molecular subtypes (12 studies)) (Table 5). Moreover, the estimated pooled RR for all studies showed a significantly increased risk of E-cad expression loss in TNBC than in the other molecular subtypes (TNBC vs. non-TNBC: RR = 1.73, 95% CI = 1.38–2.16; TNBC vs. luminal type: RR = 1.81, 95% CI = 1.19–2.75; TNBC vs. luminal A: RR = 3.45, 95% CI = 2.79–4.26; TNBC vs. luminal B: RR = 2.41, 95% CI = 1.49–3.90; TNBC vs. HER2-enriched: RR = 1.95, 95% CI = 1.24–3.07; all *P* < 0.010; Figs. 4 and 5).

**Table 5:** Pooled analysis of E-cad expression between molecular subtypes of breast cancer

Comparisons	No. of studies	Molecular subtypes	No. of patients	E-cad expression		*χ* ^2^ (*P* value)
				Negative/low	Positive	
1	4^a^ [11, 24, 25]	Luminal A	523	97	426	137.90 (0.000)
		Luminal B	305	74	231	
		HER2-enriched	208	57	151	
		TNBC	325	180	145	
2	7^a^ [11, 17, 24, 25, 27, 28]	Luminal A/B	2140	552	1588	86.34 (0.000)
		HER2-enriched	545	145	400	
		TNBC	744	324	420	
3	12^a^ [11, 12, 16, 17, 22–28]	Non-TNBC	5254	1909	3345	120.46 (0.000)
		TNBC	1273	676	597	

*TNBC* triple-negative breast cancer

^a^Including our retrospective study

#### Subgroup analysis

By grouping studies according to the publication year, geographic location, sample size, tissue processing, different IHC antibodies, cutoff value, prevalence of E-cad expression, or NOS score, the subgroup analysis showed lower heterogeneities and yielded similar associations in the overall analysis (Table 6). In geographic location subgroups, the pooled analyses of the studies conducted in Europe yielded a lower statistic of between-study heterogeneity (*I* ^2^ = 70%) than all studies (*I* ^2^ = 91%), while, if further excluding the study [28] with the lowest effect size, an *I* ^2^ statistic value of 22% was even observed. Interestingly, the recruited period subgroup analytical results revealed that much lower heterogeneities were observed between studies with recruited period before 2000 (*I* ^2^ = 10%) or after 2006 (*I* ^2^ = 0%), compared with the studies of patients recruited from 2000 to 2006 (*I* ^2^ = 91%). Moreover, the subgroup analyses also found that no statistical heterogeneities were observed between the studies those recruited a large sample size of ≥1000 (*I* ^2^ = 0%), employed 4A2C7 antibody with whole slide section tissue processing (*I* ^2^ = 38%), selected a cutoff of 100 H-score (*I* ^2^ = 0%), or reached a similar or uniform prevalence of E-cad expression of >64% (exclude the study [28] with the highest prevalence) (*I* ^2^ = 40%) or ≤50% (*I* ^2^ = 0%).

**Table 6:** The results of subgroup analyses

Variables	No. of studies	Pooled risk ratio (95% CI)	*I* ^*2*^ statistic, %	χ^2^ *P* value for heterogeneity	Analytical model
Publication year					
Before 2010	5 [12, 16, 22–24]	1.92 (1.30–2.83)	94	<0.001	REM
After 2011	7^a^ [11, 17, 25–28]	1.58 (1.15–2.18)	89	<0.001	REM
Geographic location					
Asia	7^a^ [11, 16, 17, 24–26]	2.21 (1.66–2.94)	86	<0.001	REM
Europe	5 [12, 22, 23, 27, 28]	1.26 (1.06–1.51)	70	0.010	REM
Europe (exclude the lowest effect size [28])	4 [12, 22, 23, 27]	1.29 (1.20–1.39)	22	0.280	FEM
Recruited period					
Before 2000	4 [17^b^, 22, 23 ^b^, 27]	1.29 (1.20–1.39)	10	0.340	FEM
2000 to 2006	4 [16, 24–26]	2.08 (1.40–3.08)	91	<0.001	REM
After 2006	2^a^ [11]	2.77 (2.22–3.47)	0	0.700	FEM
Sample size (*n*, median)					
≥452	6 [16, 17, 22, 23, 27, 28]	1.38 (1.07–1.78)	90	<0.001	REM
<452	6^a^ [11, 12, 24–26]	2.35 (1.45–3.81)	90	<0.001	REM
≥1000	2 [22, 27]	1.27 (1.17–1.38)	0	0.420	FEM
Tissue processing					
TMA	7 [11, 12, 17, 22, 23, 27, 28]	1.49 (1.13–1.95)	90	<0.001	REM
Whole slide section	5^a^ [16, 24–26]	2.12 (1.49–3.04)	89	<0.001	REM
IHC antibodies					
NCH-38 antibody	2 [11, 16]	2.49 (1.96–3.15)	64	0.090	REM
HECD-1 antibody	2 [12, 22]	1.50 (0.91–2.48)	71	0.060	REM
HECD-1 antibody	2 [12, 22]	1.27 (1.15–1.40)	71	0.060	FEM
4A2C7 antibody	3^a^ [23, 24]	2.29 (1.00–5.25)	94	<0.001	REM
4A2C7 antibody (only whole slide section)	2^a^ [24]	3.38 (2.47–4.62)	38	0.210	FEM
Cutoff					
10%	3 [11, 25, 26]	1.85 (1.15–2.98)	92	<0.001	REM
30 and 50%	6^a^ [12, 16, 23, 24, 28]	1.72 (1.02–2.90)	93	<0.001	REM
100 H-score	2 [22, 27]	1.27 (1.17–1.38)	0	0.420	FEM
Prevalence of E-cad expression (%, median)					
>64%	6 ^a^ [11, 12, 17, 24, 28]	2.02 (1.22–3.36)	87	<0.001	REM
>64% (exclude the highest prevalence [28])	5 ^a^ [11, 12, 17, 24]	2.74 (2.31–3.25)	40	0.150	FEM
≤64%	6 [16, 22, 23, 25–27]	1.47 (1.21–1.79)	88	<0.001	REM
≤50%	4 [22, 23, 26, 27]	1.27 (1.18–1.36)	0	0.880	FEM
NOS score					
7	5 ^a^ [16, 22, 26, 27]	1.53 (1.20–1.96)	90	<0.001	REM
5 and 6	7 [11, 12, 17, 23–25, 28]	1.82 (1.21–2.72)	90	<0.001	REM

*CI* confidence interval, *REM* random-effects model, *FEM* fixed-effects model

^a^Including our retrospective study

^b^Most half of recruited period before 2000

#### Sensitivity analysis and publication bias

**Fig. 6:**
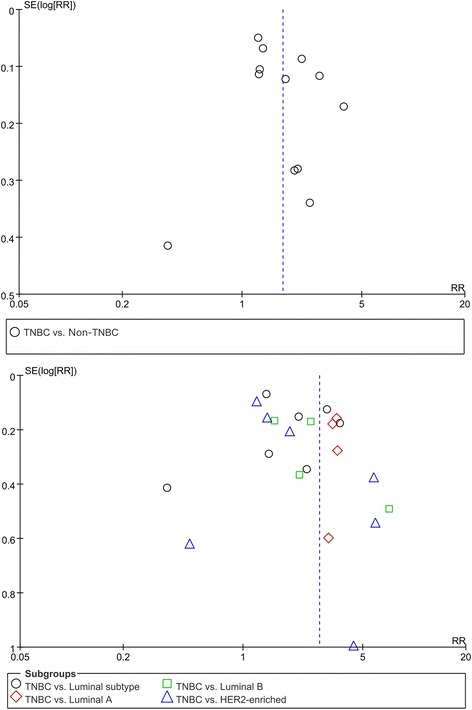
Funnel plot of comparison of E-cad expression loss between TNBC and non-TNBCs. *TNBC* triple-negative breast cancer

We conducted a sensitivity analysis to determine the influence of individual studies on the overall effect. The meta-analysis was not dominated by any single study, and the exclusion of any one study had no effect on the results: the lowest statistics were (1) TNBC vs. non-TNBC: RR = 1.60 (95% CI = 1.30–1.98); (2) TNBC vs. luminal type: RR = 1.60 (95% CI = 1.04–2.47); (3) TNBC vs. luminal A: RR = 3.43 (95% CI = 2.72–4.31); (4) TNBC vs. luminal B: RR = 1.98 (95% CI = 1.38–2.82); and (5) TNBC vs. HER2-enriched: RR = 1.57 (95% CI = 1.08–2.28); all *P* < 0.050. As depicted by the symmetrical funnel plots, the studies on the association of E-cad expression with the molecular subtypes of breast cancer showed no publication bias (Fig. 6).

## Discussion

Metastasis and recurrence are considered primary contributors to treatment failure in breast cancer. EMT is one of the mechanisms that enhance the invasive and migratory capacity of malignant cells, and it is associated with the loss of E-cad expression or function [11]. Studies have confirmed that a loss of E-cad expression in breast cancer is closely associated with invasion and metastasis [14, 15]. ER, PR, HER2, and Ki67 have all been shown to be important prognostic indicators for breast cancer. Based on the ER, PR, HER2, and Ki67 expression phenotype, molecular subtypes can be used to determine strategies for the use of hormone therapy, chemotherapy, and targeted therapy. TNBC involves the deficient expression of ER, PR, and HER2 and does not benefit from endocrine therapy or treatment with trastuzumab [30]; moreover, TNBC is prone to recurrence and metastasis after treatment even when diagnosed at an early stage [31, 32]. Therefore, finding effective therapeutic targets for TNBC is a major focus of breast cancer research.

Studies have found that TNBC is significantly associated with a loss of E-cad expression, which may explain the aggressive invasion and metastasis associated with this tumor subtype [12, 13]. However, the expression of E-cad in early-stage IDCs with a TNBC phenotype is not clear. Therefore, this retrospective study was specifically and narrowly designed to address this issue. The results show that the rate of E-cad expression loss in TNBC was significantly higher than that in non-TNBC subtypes in early-stage IDCs. Moreover, we performed a meta-analysis to collect comprehensive evidence on differences in E-cad expression between TNBC and non-TNBC subtypes of invasive non-lobular breast cancers. Similar to the results of the retrospective study, the results of the meta-analysis suggested that invasive non-lobular TNBC tumors had an approximately twofold greater tendency to present the E-cad expression loss phenotype compared with non-TNBCs.

The loss of E-cad expression or function plays a pivotal role in the process of malignant change and in the development of the invasive capacity of epithelial cells [33]. In this analysis of 156 patients with early-stage IDC, we found that the rate of E-cad expression loss of the 38 TNBC tumors was 31.6%, which was significantly higher than that of the non-TNBC tumors. Accordingly, E-cad expression loss may be a molecular mechanism promoting invasion and metastasis in TNBC cells, which would be consistent with the published results showing that E-cad expression loss is predictive of the high recurrence and mortality rates associated with TNBC tumors [17, 26]. A logistic regression analysis showed that the status of ER, PR, HER2, and Ki67 expression were not independent factors influencing the loss of E-cad expression, but the specific molecular subtype of the tumors, which is defined based on a combination of the phenotypic expression of these four markers, was a significant factor. Therefore, we speculated that the ER-, PR-, and HER2-deficient phenotype of TNBC tumors were a key factor in the determination of the E-cad-expression phenotype. Importantly, a loss of E-cad expression may be mainly attributed to the absence of ER and HER2 signaling. Studies have confirmed that ER levels can affect the invasive and metastatic phenotype of breast cancers by regulating the expression of E-cad via the ER-metastasis-associated protein MTA3-Snail-E-cad signaling pathway [34, 35]. The mechanism by which HER2 might regulate E-cad expression is not clear. A genomic analysis found that HER2 gene mutations were frequently present in relapsed invasive lobular breast cancers with a classic E-cad gene mutation [36]. In addition, a loss of E-cad expression is a key indicator of EMT. However, recent studies have found that the loss of E-cad expression is not a prerequisite for the initiation of EMT induced by HER2 signaling, but is actually a subsequent molecular event occurring after EMT [37, 38]. Therefore, these findings suggest a sophisticated association between HER2 signaling and E-cad expression, which requires the design of additional experiments to reveal the complex mechanism of interaction between these two molecules.

The results of the meta-analysis of the association of E-cad expression with specific molecular subtypes of invasive non-lobular breast cancer also show a relation between ER, HER2 signaling, and E-cad expression. The results from the overall meta-analysis and the sensitivity analysis all show that TNBC tumors have higher risk of developing E-cad expression loss than other tumor types and this risk is progressively decreased in luminal A, luminal B, and HER2-enriched tumors. These findings suggest that the ER- and PR-positive molecular phenotype in luminal A tumors present a normal ER-E-cad axis for upregulating and stabilizing E-cad expression, precluding the loss of E-cad expression [34, 35], while the combination of the ER and PR positive or negative and HER2-positive expression phenotypes in luminal B and HER2-enriched tumors may involve the combined effects of ER and HER2 on E-cad expression, resulting in a variable HER2-E-cad signaling pathway. Accordingly, we believe that our meta-analysis will provide useful information for further research in invasive non-lobular breast cancer.

In the meta-analysis, significant heterogeneity was observed between the included studies. For investigating the source of high between-study heterogeneity, subgroup analysis was performed. Finally, the subgroup analytical results yielded unchanged associations between E-cad expression and molecular subtypes of invasive non-lobular breast cancer in different stratifications and suggested that geographic location, recruited period, sample size, IHC antibodies and tissue processing, cutoff value, and prevalence of E-cad expression could be the potential sources of the heterogeneities between the included studies of the meta-analysis, while publication year and NOS score might have no impact on the between-study heterogeneity of this meta-analysis.

There were some limitations in the meta-analysis. Firstly, except for invasive non-lobular breast cancers, the main goal of the meta-analysis was to investigate E-cad expression between molecular subtypes of early-stage IDCs; however, because there are no published studies about the topic, no individual data about early-stage IDC were present in the included studies, and not to connect with the authors of the included studies for obtaining the raw data, the main evidence for E-cad expression between the molecular subtypes of early-stage IDCs was only from our retrospective study. Furthermore, semi-quantitative IHC detection may affect the precision of the results and between-studies heterogeneity due to differences in the primary antibodies, IHC staining protocols, evaluation standards, and cut﻿off values for E-cad expression used, as well as differences in the surrogate markers used for the determination of molecular subtypes. Finally, we believe there could be potential language and publication biases in the meta-analysis because we only sought published studies written in English and Chinese. Thus, we suggest that the results of the meta-analysis should be interpreted cautiously.

## Conclusion

In sum, our results confirm that the E-cad expression phenotypes were closely related to molecular subtypes and further studies are needed to clarify the underlying mechanism. Early-stage IDCs or invasive non-lobular breast cancers with TNBC molecular profiles had a higher risk for the loss of E-cad expression than tumors with non-TNBC molecular subtypes. E-cad expression is emerging as an important factor in the invasion and metastasis of TNBC tumors. We expect that E-cad expression may serve as a reliable tool for early and accurate predictions of invasion and metastasis of TNBC and may be a potential therapeutic target for treating invasive non-lobular breast cancer.

## Additional file

Additional file 1: Quality assessments of included studies [11, 16, 17, 22–28]. (DOCX 16 kb)
