# Supplementary material for: E-cadherin expression phenotypes associated with molecular subtypes in invasive non-lobular breast cancer: evidence from a retrospective study and meta-analysis
Source: World J Surg Oncol. 2017 Aug 1;15:139. doi: 10.1186/s12957-017-1210-8 (PMC5539617; doi:10.1186/s12957-017-1210-8)
Supplement: Additional file 1: — Quality assessments of included studies [11, 16, 17, 22–28]. (DOCX 16 kb) [file 12957_2017_1210_MOESM1_ESM.docx]

**Table S1.** Quality assessments of included studies.

| Reference | Country | 1 | 2 | 3 | 4 | 5A | 5B | 6 | 7 | 8 | Total |
| --- | --- | --- | --- | --- | --- | --- | --- | --- | --- | --- | --- |
| Aleskandarany et al 2014 [27] | UK | 1 | 1 | 1 | 1 | 1 | 0 | 1 | 1 | 0 | 7 |
| Choi et al 2013 [11] | Korea | 1 | 1 | 1 | 1 | 0 | 1 | 0 | 1 | 0 | 6 |
| Jeong et al 2012 [17] | Korea | 1 | 1 | 1 | 1 | 0 | 1 | 0 | 1 | 0 | 6 |
| Kashiwagi et al 2010 [16] | Japan | 1 | 1 | 1 | 1 | 1 | 1 | 0 | 1 | 0 | 7 |
| Liu et al 2013 [25] | China | 1 | 1 | 1 | 1 | 0 | 1 | 0 | 1 | 0 | 6 |
| Mahler-Araujo et al 2008 [11] | UK | 1 | 1 | 1 | 1 | 0 | 0 | 0 | 1 | 0 | 5 |
| Pang et al 2013 [26] | China | 1 | 1 | 1 | 1 | 1 | 1 | 0 | 1 | 0 | 7 |
| Pomp et al 2015 [28] | Switzerland | 1 | 1 | 1 | 1 | 0 | 1 | 0 | 1 | 0 | 6 |
| Rakha et al 2006 [22] | UK | 1 | 1 | 1 | 1 | 0 | 1 | 1 | 1 | 0 | 7 |
| Sarrio et al 2008 [23] | Spain | 1 | 1 | 1 | 1 | 0 | 0 | 0 | 1 | 0 | 5 |
| Wu et al 2009 [24] | China | 1 | 1 | 1 | 1 | 0 | 1 | 0 | 1 | 0 | 6 |

1: with independent validation; 2: consecutive or obviously representative series of cases; 3: community controls; 4: first occurrence of outcome; 5A: study controls for age; 5B: study controls for any additional factor (tumor stage); 6: structured interview where blind to case/control status; 7: same method of ascertainment for cases and controls; 8: same Non-Response Rate for both groups.
